# Atg5 in microglia regulates sex-specific effects on postnatal neurogenesis in Alzheimer’s disease

**DOI:** 10.1038/s41514-025-00209-0

**Published:** 2025-03-16

**Authors:** Ellen Walter, Gabrielle Angst, Justin Bollinger, Linh Truong, Elena Ware, Eric S. Wohleb, Yanbo Fan, Chenran Wang

**Affiliations:** 1 Department of Cancer Biology, University of Cincinnati College Medicine, Cincinnati, OH USA; 2 Department of Pharmacology & Systems Physiology, University of Cincinnati College Medicine, Cincinnati, OH USA; 3 Present Address: Department of Radiation Oncology, Ohio State Comprehensive Cancer Center, Arthur G. James Cancer Hospital and Richard J. Solove Research Institute and College of Medicine at The Ohio State University, Columbus, OH USA; 4 Present Address: Center for Cancer Metabolism, James Comprehensive Cancer Center at The Ohio State University, Columbus, OH USA

**Keywords:** Neurodegenerative diseases, Neuroscience, Diseases of the nervous system, Neural ageing, Neurological disorders, Dementia, Alzheimer's disease, Autophagy

## Abstract

Female Alzheimer’s disease (AD) patients display greater cognitive deficits and worse AD pathology as compared to male AD patients. In this study, we found that conditional knockout (cKO) of *Atg5* in female microglia failed to obtain disease-associated microglia (DAM) gene signatures in familiar AD mouse model (5xFAD). Next, we analyzed the maintenance and neurogenesis of neural stem cells (NSCs) in the hippocampus and subventricular zone (SVZ) from 5xFAD mice with *Atg5* cKO. Our data indicated that *Atg5* cKO reduced the NSC number in hippocampus of female but not male 5xFAD mice. However, in the SVZ, *Atg5* cKO only impaired NSCs in male 5xFAD mice. Interestingly, female 5xFAD;*Fip200* cKO mice and 5xFAD;*Atg14* cKO mice did not show NSC defects. These autophagy genes cKO 5xFAD mice exhibited a higher neurogenesis activity in their SVZ. Together, our data indicate a sex-specific role for microglial *Atg5* in postnatal neurogenesis in AD mice.

## Introduction

Women have a greater lifetime risk of developing Alzheimer’s disease (AD) compared with men, and two thirds of AD patients are females^1,2^. The higher prevalence of AD in women might be attributed to their longer life expectancy as some previous studies suggest that the risk of developing AD might not have a significant difference between genders after adjustment for age^3,4^. Even though aging is the biggest known risk factor for AD up to now, the primary drivers of biological sex by gonadal hormones and sex chromosomes also influences AD risk and progression. Females have sharply reduced neuroprotective estrogen production after menopause, which might account for more female AD patients and heavier burdens of Aβ plaque and tau pathology^2,5^. A very recent study suggests that X-linked gene *Tlr7* determines the sex differences in the demyelination in AD^6^. Despite the recent progress, the factors and their roles in influencing sex-dependent outcomes in AD progression remain elusive in the field.

Postnatal neurogenesis, the generation of new neurons after birth, exists in the subgranular zone (SGZ) of the dentate gyrus (DG) in the hippocampus and the subventricular zone (SVZ) of lateral ventricle^7–12^. Postnatal neurogenesis is driven by neural progenitor/stem cells (NSCs) in these regions and is important for the maintenance and reorganization of the existing circuitry in homeostasis and brain repair after injuries^13–18^. Impaired postnatal neurogenesis, loss of synapses, and increased microglial reactivity are features of the aged brain in both humans and mice^19^. Abnormalities in postnatal neurogenesis also have been linked to the early onset of neurodegenerative disorders such as AD^20,21^. It has been shown that blocking postnatal neurogenesis exacerbates cognitive impairment in a preclinical AD mouse model^22^. The sex differences for postnatal neurogenesis have been noticed in rodent under physiological or stressed conditions^23–25^ while postnatal neurogenesis is expected to contribute to the sex differences in cognitive function of AD patients^26^. However, the regulation and mechanism of postnatal neurogenesis in male and female AD brain are still largely unexplored.

Autophagy is a self-degradation process of cytoplasmic content or organelles to maintain homeostasis^27,28^. Our previous studies identify the functions of autophagy in NSCs to maintain their self-renewal and neurogenesis via both cell-autonomous and non-cell-autonomous mechanisms in development and aging^29–32^. Autophagy activity in neural cells, including neurons, NSCs, and microglia, decreases with aging and in late stages of neurodegenerative diseases^31,33,34^. Sex differences in baseline autophagy levels and stimulus-mediated autophagic flux have been identified in both mice and humans, with females showing reduced autophagic activity compared to males throughout their lifetime^5,35^. Contextually, both estradiol and progesterone enhance the gene expression of autophagy related gene 3 (*Atg3*), *Atg5*, and *Beclin-1 (Becn-1)* in mammary epithelial cells^36^. Potential estrogen or androgen receptor binding sites have been identified in the promoter regions of two-thirds of genes encoding autophagy proteins, and 84% of core autophagy genes can be transcriptionally regulated by sex steroid receptors^37^. Sex-specific functions of autophagy and autophagy genes have been described in different human diseases. Single nucleotide polymorphisms of *Atg16l1* gene are associated with increased chances of ankylosing spondylitis and Crohn’s disease in females^38,39^. Becn-1 is phosphorylated by AMPK to enhance autophagy in prefrontal cortex of female schizophrenia patients, which might be responsible for the better cognitive outcome compared to male patients^40^. The functions and mechanisms of the changes in autophagy genes or autophagy proteins are gaining attention for studies of sex-dimorphous in AD. However, the mechanisms whereby dysfunctional autophagy contributes to sex differences in AD progression remain poorly understood.

Microglia are yolk sac-derived macrophages that colonize the central nervous system during early embryonic development. They comprise 10% of brain cells and serve as the primary defense against insults^41,42^. Microglia are highly dynamic modulators in brain development and diseases^43–45^ including the participation in synaptic remodeling, neurogenesis, elimination of unwanted neurons and debris^46^. Microglia heterogeneity is involved in their maturation and the progression of diseases^47–49^. Studies indicate that disease-associated microglia (DAM), defined by a small set of altered genes by single cell RNA-sequencing, are linked to AD, and DAM-like heterogeneity has been characterized in other neurodegenerative diseases^50–53^. Sex differences in gene expression and cellular functions are evident in adult microglia^54,55^ and more obvious in aged brains and AD^56–58^. For example, microglia from both sexes increase their phagocytosis of neural debris with aging while aged female microglia performed better than aged male microglia. However, aged female microglia could not adapt their phagocytosis following an inflammatory challenge. Likewise, in a Tau-related AD mouse model, Kodama et al. show that deletion of microRNAs in male microglia leads to transcriptome changes toward DAM and increases tau pathology^59^. These findings suggest that sex differences in aged microglia may play a unique role in AD^60^.

Recently, the functions of autophagy in microglia in AD progression have been revealed by several studies. Microglial Atg5 and RUBCN/Rubicon protect 5xFAD mice from Aβ accumulation, neuroinflammation, and neurodegeneration^61^. Mechanistically, canonical autophagy-independent functions of LC3-associated endocytosis enables removal of Aβ and ameliorates pathology in these murine AD models. Using microglia specific *Atg7* cKO AD mice, a recent study revealed impaired DAM gene signatures with increased microglial senescence, which causes more severe neurodegeneration in 5xFAD mice^62^. However, the functions and mechanisms of autophagy in microglia to regulate AD neurogenesis at early disease stages remain unclear. Our previous results indicate that ablation of *Atg5* in microglia impairs the maintenance and neurogenesis of NSCs in the hippocampus of young adult female, but not male, 5xFAD mice^63^. In the present study, we compared the expression of DAM gene signature in hippocampal microglia from male and female 5xFAD *Atg5* cKO mice. We found that *Atg5* deletion in female microglia significantly reduced their expression of DAM genes, which coincided with the early degeneration of NSCs in female 5xFAD hippocampus. We extended our research to a later stage of AD progression in 5xFAD mice lacking microglial *Atg5*, and in female 5xFAD mice with microglia-specific ablation of two additional autophagy essential genes (*Fip200* and *Atg14*). We observed impaired NSC maintenance only in the hippocampus of female 5xFAD *Atg5* cKO mice. Our results support a sex-specific role for microglial *Atg5* in the protection of NSCs in 5xFAD through regulating DAM gene signatures.

## Results

### Atg5 deficiency in female, but not in male, microglia inhibited the induction of disease associated microglia (DAM) gene signatures in the hippocampus of 5xFAD mice

**Fig. 1:**
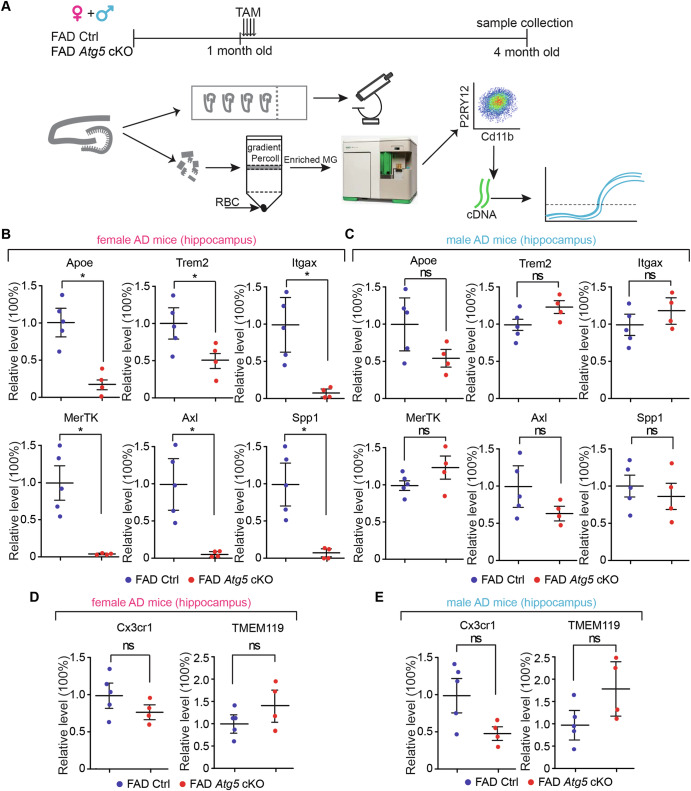
Atg5 deletion in microglia reduced the mRNA level of DAM genes in hippocampus of 4-month-old female 5xFAD mice. **A** Schematic depiction of experimental design for characterization of microglia from male and female 5xFAD Ctrl and 5xFAD *Atg5* cKO mice at 4-month-old. Mean ± SE of the relative mRNA levels of Apoe, Trem2, Itgax, MerTK, Axl, and Spp1 in microglia from the hippocampus of female (**B**) and male (**C**) 5xFAD Ctrl and 5xFAD *Atg5* cKO mice at 4-month-old. Mean ± SE of the relative mRNA levels of Cx3cr1 and Tmem119 in microglia from the hippocampus of female (**D**) and male (**E**) 5xFAD Ctrl and 5xFAD *Atg5* cKO mice at 4-month-old. Female 5xFAD Ctrl = 5 mice and 5xFAD *Atg5* cKO = 4 mice; male 5xFAD Ctrl = 5 mice and male 5xFAD *Atg5* cKO = 4 mice. All statistical analysis done was Student’s t-test. ns no significance, *: p < 0.05.

**Fig. 2:**
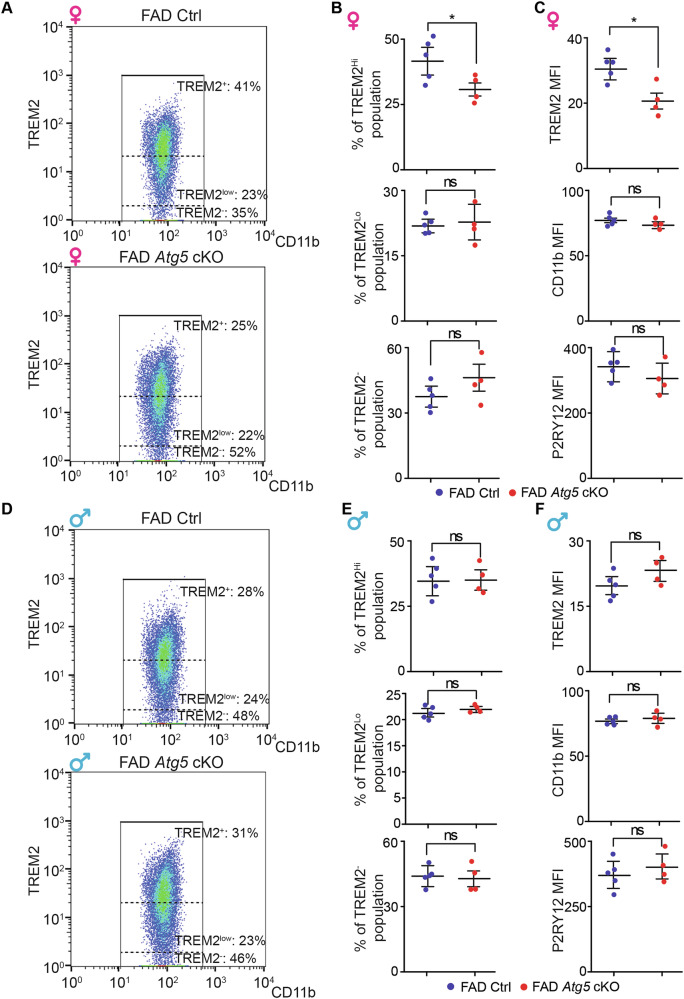
Atg5 deletion in microglia reduced the surface expression of TREM2 in hippocampus of 4-month-old female 5xFAD mice. **A** Flow cytometry analyses of the surface expression of CD11b and TREM2 on isolated hippocampal microglia from female 5xFAD Ctrl and 5xFAD *Atg5* cKO mice at 4-month-old. **B** Mean ± SE of the percentage of TREM2 high (top), TREM2 low (middle), and TREM2 negative (bottom) microglia of all microglia from female 5xFAD Ctrl and 5xFAD *Atg5* cKO mice at 4-month-old. **C** Mean ± SE of the MFI of TREM2 (top), CD11b (middle), and P2RY12 (bottom) on isolated microglia from female 5xFAD Ctrl and 5xFAD *Atg5* cKO mice at 4-month-old. **D** Flow cytometry analyses of the surface expression of CD11b and TREM2 on isolated hippocampal microglia from male 5xFAD Ctrl and 5xFAD *Atg5* cKO mice at 4-month-old. **E** Mean ± SE of the percentage of TREM2 high (top), TREM2 low (middle), and TREM2 negative (bottom) microglia of all microglia from male 5xFAD Ctrl and 5xFAD *Atg5* cKO mice at 4-month-old. **F** Mean ± SE of the MFI of TREM2 (top), CD11b (middle), and P2RY12 (bottom) on isolated microglia from male 5xFAD Ctrl and 5xFAD *Atg5* cKO mice at 4-month-old. Female 5xFAD Ctrl = 5 mice and 5xFAD *Atg5* cKO = 4 mice; male 5xFAD Ctrl = 5 mice and male 5xFAD *Atg5* cKO = 4 mice. All statistical analysis done was Student’s t-test. ns no significance, *: p < 0.05.

Our earlier work indicates a protective function of *Atg5* in female, but not in male microglia for postnatal neurogenesis in 5xFAD hippocampus at 4-month-old^63^. To further examine this, we manipulated microglial *Atg5* using an inducible Cre-lox approach by crossing *Atg5* flox/flox; CX3CR1^CreERT2^ ^64^ with 5xFAD transgenic mice^65^. After intraperitoneal injection of tamoxifen (TAM, 1 mg each injection for 4 times) at 1-month-old, we isolated microglia by FACS^66^ from the hippocampus of 4-month-old control (Atg5 flox/flox), 5xFAD Ctrl (5xFAD;Atg5 flox/flox) and 5xFAD *Atg5* cKO (5xFAD;Atg5 flox/flox; CX3CR1^CreERT2^) mice for downstream analysis (Fig. 1A and S1A). Our data indicated that at 4-month-old, the mean fluorescent intensity (MFI) of P2RY12 was comparable in microglia isolated from female 5xFAD Ctrl mice and matched female control mice (Fig. S1B and S1C). This suggested that our sorting did not exclude microglia in AD brains, which might reduce their P2RY12 level at a much later disease stage^53^. We found that deletion of *Atg5* in female microglia significantly decreased the expression levels of DAM genes *Apoe*, *Trem2*, *Itgax*, *Mertk*, *Axl*, and *Spp1* (Fig. 1B). Loss of *Atg5* in male microglia had no impact on the expression of these genes in the 5xFAD hippocampus (Fig. 1C). Knockout of microglial *Atg5* did not significantly affect the levels of microglia homeostasis markers *Cx3cr1* and *Tmem119* in the hippocampus of either sex (Fig. 1D, E). Using flow cytometry, we analyzed the surface receptor levels of TREM2, CD11b, and P2RY12 on hippocampal microglia from both male and female 5xFAD mice with- or without *Atg5* ablation. The proportion of TREM2^high^ microglia significantly decreased in female 5xFAD *Atg5* cKO mice at 4-month-old but the percentage of TREM2^low^ and TREM2^neg^ microglia did not show a significant difference (Fig. 2A, B). Consistent with RT-qPCR results, we found a significant reduction in the MFI of TREM2 on hippocampal microglia in female 5xFAD *Atg5* cKO mice (Fig. 2A, C). No differences were detected in CD11b or P2RY12 MFI (Fig. 2C). In contrast to female microglia, loss of *Atg5* in male 5xFAD mice had no effect on the proportion of TREM2^high^, TREM2^low^, and TREM2^neg^ microglia (Fig. 2D, E) or MFI of TREM2, CD11b or P2RY12 in the hippocampus (Fig. 2F). Together, these data indicated that *Atg5* in female, but not in male microglia was critical for the acquisition of DAM gene signatures in the 5xFAD hippocampus.

### Atg5 deficiency in female, but not male, microglia impaired the maintenance of hippocampal NSC of 8-month-old 5xFAD mice

**Fig. 3:**
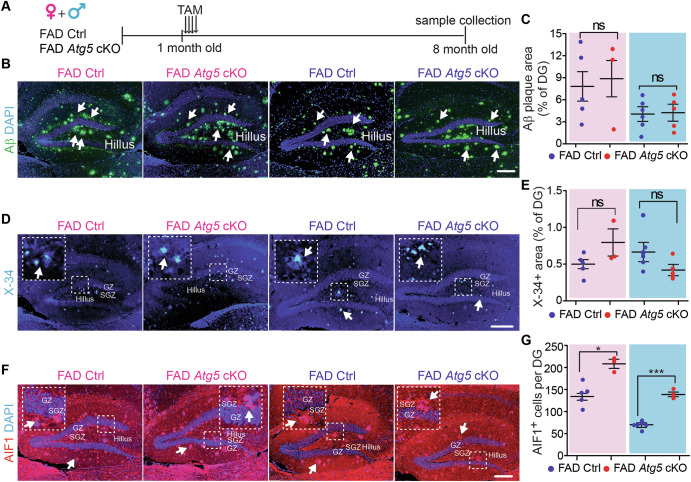
Atg5 deletion in microglia did not affect Aβ plaques formation but increased microglia number in DG of 8-month-old 5xFAD mice. **A** Schematic depiction of experimental design for 8-month-old samples from male and female 5xFAD Ctrl and 5xFAD *Atg5* cKO mice. **B** Immunofluorescence (IF) of Aβ and DAPI in the hippocampus of 5xFAD Ctrl and 5xFAD *Atg5* cKO mice at 8-month-old. Arrows indicated Aβ plaques. **C** Mean ± SE of the percentage of Aβ plaques coverage of the total DG area in 5xFAD Ctrl and 5xFAD *Atg5* cKO mice. **D** X-34 staining in the hippocampus of 5xFAD Ctrl and 5xFAD *Atg5* cKO mice. X-34 agglomerates were indicated by arrows. Boxed areas were shown in detail as insets. **E** Mean ± SE of the percentage of X-34 coverage of the total DG area in 5xFAD Ctrl and 5xFAD *Atg5* cKO mice. **F** IF of AIF1 and DAPI in the hippocampus of 5xFAD Ctrl mice and 5xFAD *Atg5* cKO mice. Arrows indicated microglia. Boxed areas were shown in detail as insets. **G** Mean ± SE of the number of AIF1^+^ cells in the DG of 5xFAD Ctrl and 5xFAD *Atg5* cKO mice. GZ granular zone, SGZ subgranular zone. Female 5xFAD Ctrl = 5 mice and 5xFAD *Atg5* cKO = 3 mice; male 5xFAD Ctrl = 6 mice and male 5xFAD *Atg5* cKO = 5 mice. The Student’s t-test was used for statistical analysis. ns no significance, *: p < 0.05, ***: p < 0.001. Bar = 100 μm.

**Fig. 4:**
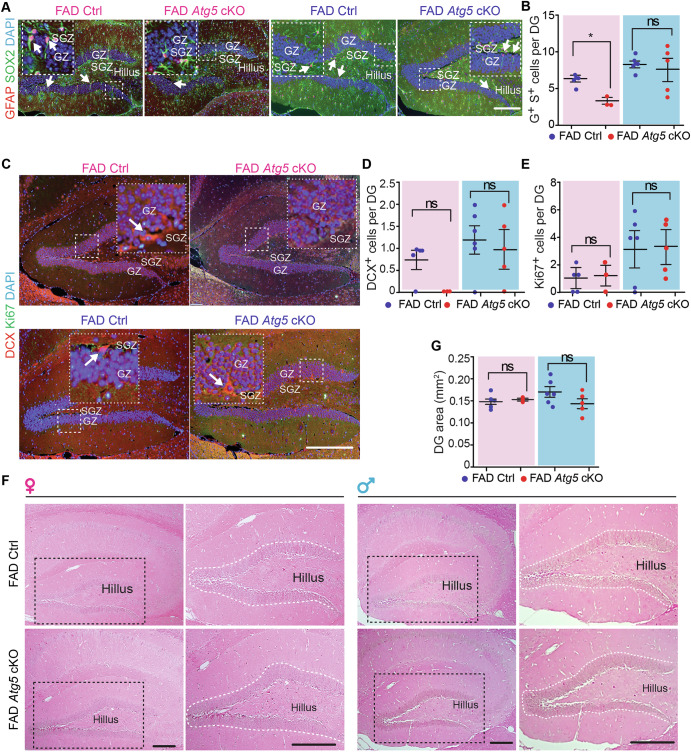
Atg5 deletion in female, but not male microglia impaired NSC maintenance in DG of 8-month-old 5xFAD mice. **A** IF of SOX2, GFAP, and DAPI in the DG of male and female 5xFAD Ctrl and 5xFAD *Atg5* cKO mice. Arrows indicated NSCs. Boxed areas were shown in detail as insets. **B** Mean ± SE of the number of NSCs in the DG of 5xFAD Ctrl and 5xFAD *Atg5* cKO mice. **C** IF of DCX, Ki67, and DAPI in the DG of 5xFAD Ctrl and 5xFAD *Atg5* cKO mice. Arrows indicated DCX^+^ neuroblast. Boxed areas were shown in detail as insets. Mean ± SE of the number of DCX^+^ neuroblast (**D**) and Ki67^+^ cells (**E**) in the DG of 5xFAD Ctrl and 5xFAD *Atg5* cKO mice. **F** H&E staining of hippocampus of 5xFAD Ctrl and 5xFAD *Atg5* cKO mice. Enlarged images of DG and CA1 were shown in the middle and on the right. **G** Mean ± SE of the DG area of 5xFAD Ctrl and 5xFAD *Atg5* cKO mice. CA1 Cornu Ammonis 1, GZ granular zone, SGZ subgranular zone. Female 5xFAD Ctrl = 5 mice and 5xFAD *Atg5* cKO = 3 mice; male 5xFAD Ctrl = 6 mice and male 5xFAD *Atg5* cKO = 5 mice. The Student’s t-test was used for statistical analysis. ns no significance, *: p < 0.05. Bar = 100 μm.

Next, we compared the Atg5 level in microglia from 5xFAD Ctrl mice and 5xFAD *Atg5* cKO mice at 8-month-old. Our data indicated that the number of Atg5^+^ microglia and the levels of Atg5 MFI were significantly reduced in hippocampus from TAM-treated 5xFAD *Atg5* cKO mice from both sexes (Fig. S2A–C). Next, we examined the Aβ plaques, microglia, and NSCs in hippocampus of these mice from both sexes at 8-month-old (Fig. 3A). We performed immunostaining to detect Aβ plaques and observed a significant amount of Aβ plaques deposition in the hippocampus of 5xFAD Ctrl mice and 5xFAD *Atg5* cKO mice at 8-month-old (Fig. 3B). Loss of microglial *Atg5* had no effect on the total area of Aβ plaque deposition in DG (Fig. 3B, C) which was further confirmed by using X-34 staining, another approach to visualize Aβ plaques^67^ (Fig. 3D, E). These results suggested that *Atg5* deficiency in microglia had no impact on Aβ plaques, even at advanced disease stage. Then, we used AIF1 (allograft inflammatory factor 1, also known as IBA1) to label microglia and we found significantly more AIF1^+^ microglia in the DG of 5xFAD *Atg5* cKO mice than in 5xFAD Ctrl mice, irrespective of their sexes (Fig. 3F, G). These results suggested that *Atg5* depletion promoted the survival and/or inhibited the death of microglia in 5xFAD mice at 8-month-old. We performed double staining of GFAP (glial fibrillary acidic protein) and SOX2 (SRY (sex determining region Y)-box 2) to label postnatal NSCs with a radial glia morphology in the SGZ of the hippocampus^29,30^. We found a significant decrease in the number of GFAP^+^ SOX2^+^ NSCs only in female 5xFAD *Atg5* cKO mice (Fig. 4A, B). However, the number of hippocampal DCX (doublecortin)-positive (DCX^+^) immature neurons and Ki67^+^ proliferative cells was comparable in in both female and male 5xFAD mice, regardless of their *Atg5* status in microglia (Fig. 4C–E). Last, we quantified the area of the DG and Cornu Ammonis 1 (CA1) subregions of the hippocampus. We did not detect any effect of microglial *Atg5* cKO on the thickness of these hippocampal subregions in 5xFAD mice at 8-month-old (Fig. 4F, G). Collectively, these results suggested that *Atg5*-deficient microglia reduced hippocampal NSC content in female 5xFAD mice, but they had little or no impact on the maintenance and neurogenesis of NSCs in the hippocampus of male 5xFAD mice.

### Increased neuroblast generation in the SVZ of 8-month-old 5xFAD *Atg5* cKO mice

**Fig. 5:**
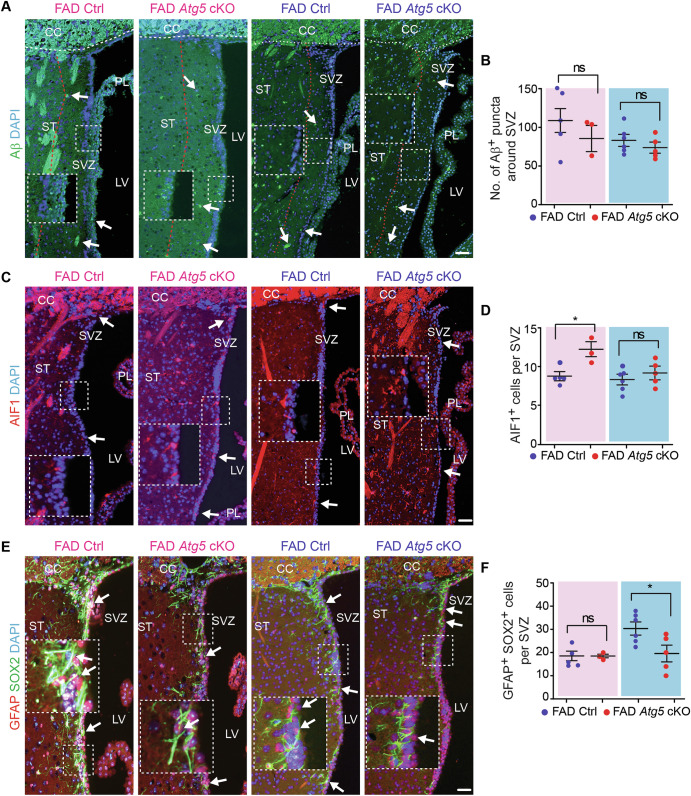
Atg5 deletion in microglia reduced NSC maintenance in SVZ of 8-month-old male AD mice. **A** IF of Aβ and DAPI in the striatum and SVZ of male and female 5xFAD Ctrl and 5xFAD *Atg5* cKO mice at 8-month-old. Arrows indicated Aβ^+^ dots. Boxed areas were shown in detail as insets. Red dotted lines indicated the regions 200 μm away from the wall of lateral ventricle. **B** Mean ± SE of the number of Aβ puncta within 200 µm distance of SVZ and striatum in 5xFAD Ctrl and 5xFAD *Atg5* cKO mice. **C** IF of AIF1 and DAPI in SVZ of 5xFAD Ctrl mice and 5xFAD *Atg5* cKO mice. Arrows indicated microglia. Boxed areas were shown in detail as insets. **D** Mean ± SE of the number of AIF1^+^ cells in SVZ of 5xFAD Ctrl and 5xFAD *Atg5* cKO mice from both sexes. **E** IF of SOX2, GFAP, and DAPI in SVZ of 5xFAD Ctrl and 5xFAD *Atg5* cKO mice. Arrows indicated NSCs. Boxed areas were shown in detail as insets. **F** Mean ± SE of the number of NSCs in SVZ of 5xFAD Ctrl and 5xFAD *Atg5* cKO mice. CC corpus callosum, LV lateral ventricle, PL plexus, ST striatum, SVZ subventricular zone. Female 5xFAD Ctrl = 5 mice and 5xFAD *Atg5* cKO = 3 mice; male 5xFAD Ctrl = 6 mice and male 5xFAD *Atg5* cKO = 5 mice. The Student’s t-test was used for statistical analysis. ns no significance, *: p < 0.05. Bar = 100 μm.

**Fig. 6:**
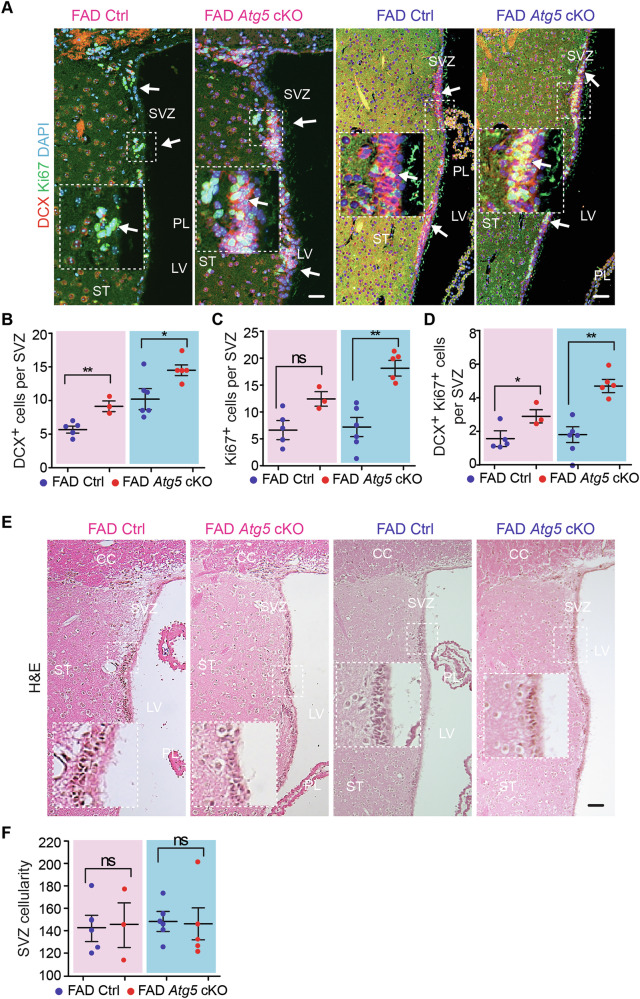
Increased neuroblasts and their proliferation in SVZ of 8-month-old male and female 5xFAD *Atg5* cKO mice. **A** IF of DCX, Ki67, and DAPI of SVZ in male and female 5xFAD Ctrl and 5xFAD *Atg5* cKO mice at 8-month-old. Arrows indicated DCX^+^ Ki67^+^ neuroblasts. Boxed areas were shown in detail as insets. Mean ± SE of the number of DCX^+^ cells (**B**), Ki67^+^ cells (**C**), and DCX^+^ Ki67^+^ cells (**D**) in SVZ of 5xFAD Ctrl and 5xFAD *Atg5* cKO mice. **E** H&E staining of SVZ in male and female 5xFAD Ctrl and 5xFAD *Atg5* cKO mice. Boxed areas were shown in detail as insets. **F** Mean ± SE of the SVZ cell number in 5xFAD Ctrl and 5xFAD *Atg5* cKO mice. CC corpus callosum, LV lateral ventricle, PL plexus, ST striatum, SVZ subventricular zone. Female 5xFAD Ctrl = 5 mice and 5xFAD *Atg5* cKO = 3 mice; male 5xFAD Ctrl = 6 mice and male 5xFAD *Atg5* cKO = 5 mice. The Student’s t-test was used for statistical analysis. ns no significance, *: p < 0.05, **: p < 0.01. Bar = 100 μm.

Alongside the hippocampus, the SVZ is capable of neurogenesis in adult mice. Thus, we examined the NSC of SVZ in 5xFAD Ctrl and 5xFAD *Atg5* cKO mice at 8-month-old. As compared to the AD hippocampus, fewer and smaller Aβ plaques were observed in the striatum (Fig. 5A). We quantified the amount of Aβ^+^ dots within an area of 200 μm from the wall of LV to striatum, covering the whole SVZ and adjacent striatum. We did not find differences in the total number of Aβ dots between 5xFAD Ctrl and 5xFAD *Atg5* cKO mice for both sexes (Fig. 5A, B). More microglia were found in SVZ of female 5xFAD *Atg5* cKO mice and no differences were detected in the SVZ microglia number of male 5xFAD mice lacking microglial *Atg5*, as compared to their respective 5xFAD Ctrl mice (Fig. 5C, D). We performed double staining of GFAP and SOX2 to label NSCs in SVZ. Our data indicated a significantly decreased number of GFAP^+^ SOX2^+^ NSCs in the SVZ of male, but not female, 5xFAD *Atg5* cKO mice at 8-month-old (Fig. 5E, F). Interestingly, the number of DCX^+^ cells in SVZ significantly increased in 5xFAD *Atg5* cKO mice from both sexes (Fig. 6A, B). The numbers of Ki67^+^ progenitors were higher in male 5xFAD *Atg5* cKO mice, however the number of DCX^+^ Ki67^+^ immature neurons increased in the SVZ of both female and male 5xFAD *Atg5* cKO mice at 8-month-old (Fig. 6A, C, D). H&E staining did not show significant difference in the number of SVZ cells between these 5xFAD mice (Fig. 6E, F). Together, these data suggested that male *Atg5*-deficient microglia impaired the maintenance of postnatal NSCs in the SVZ of 5xFAD mice while Atg5 might have a sex-independent functions in microglia in restricting the proliferation of progenitors/immature neurons in this neurogenic region.

### Loss of autophagy genes *Fip200* and *Atg14* in microglia had no effect on the maintenance or neurogenesis of NSCs in female 5xFAD mice at 8-month-old

**Fig. 7:**
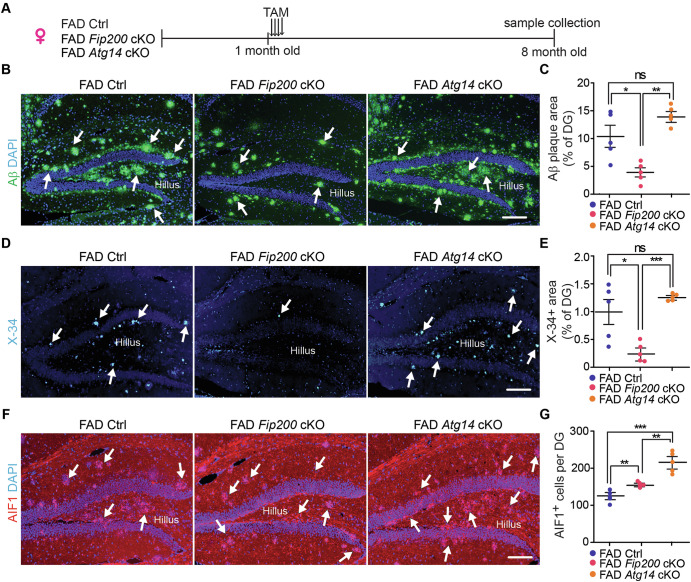
Deletion of Fip200 and Atg14 in hippocampal microglia of female 8-month-old 5XFAD mice. **A** Schematic depiction of experimental design for 8-month-old samples from female 5XFAD Ctrl, 5XFAD *Fip200* cKO, and 5XFAD *Atg14* cKO mice. **B** IF of Aβ and DAPI in the hippocampus of female 5XFAD Ctrl, 5XFAD *Fip200* cKO, and 5XFAD *Atg14* cKO mice at 8-month-old. Arrows indicated Aβ plaques. **C** Mean ± SE of the percentage of Aβ coverage of the DG area in 5xFAD Ctrl, 5xFAD *Fip200* cKO, and 5xFAD *Atg14* cKO mice. **D** X-34 staining in the DG of female 5xFAD Ctrl, 5xFAD *Fip200* cKO, and 5xFAD *Atg14* cKO mice at 8-month-old. X-34 agglomerates were indicated by arrows. **E** Mean ± SE of the percentage of X-34 coverage of the DG area in 5xFAD Ctrl, 5xFAD *Fip200* cKO, and 5xFAD *Atg14* cKO mice. **F** IF of AIF1 and DAPI in the DG of female 5xFAD Ctrl, 5xFAD *Fip200* cKO, and 5xFAD *Atg14* cKO mice. Arrows indicated microglia. **G** Mean ± SE of the number of AIF1^+^ cells in the DG of 5xFAD Ctrl, 5xFAD *Fip200* cKO, and 5xFAD *Atg14* cKO mice. 5xFAD Ctrl = 5 mice, 5xFAD *Fip200* cKO = 5 mice, and 5xFAD *Atg14* cKO = 5 mice. One-way ANOVA with a Bonferroni correction was used for statistical analysis. ns no significance, *: p < 0.05, **: p < 0.01, ***: p < 0.001. Bar = 100 μm.

**Fig. 8:**
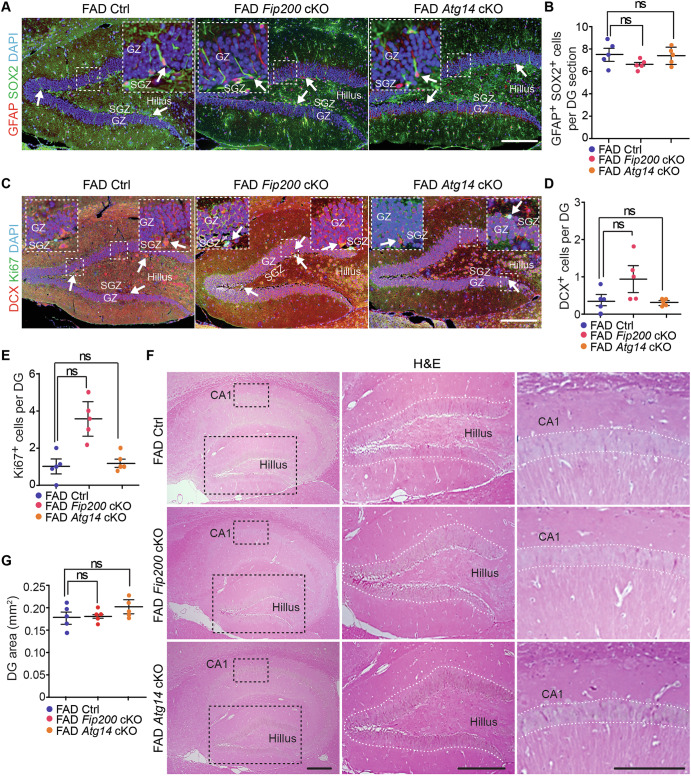
Fip200 and Atg14 deletion in microglia had no impact on the maintenance and neurogenesis of hippocampal NSC in 8-month-old female AD mice. **A** IF of SOX2, GFAP, and DAPI in the DG of female 5xFAD Ctrl, 5xFAD *Fip200* cKO, and 5xFAD *Atg14* cKO mice at 8-month-old. Arrows indicated NSCs. Boxed areas were shown in detail as insets. **B** Mean ± SE of the number of NSCs in the DG of female 5xFAD Ctrl, 5xFAD *Fip200* cKO, and 5xFAD *Atg14* cKO mice. **C** IF of DCX, Ki67, and DAPI in the DG of female 5xFAD Ctrl, 5xFAD *Fip200* cKO, 5xFAD *Atg14* cKO mice. Arrows indicated DCX^+^ neuroblast or Ki67^+^ proliferative cells. Boxed areas were shown in detail as insets. Mean ± SE of the number of DCX^+^ neuroblast (**D**) and the number of Ki67^+^ cells (**E**) in the DG of female 5xFAD Ctrl, 5xFAD *Fip200* cKO, 5xFAD *Atg14* cKO mice at 8-month-old. **F** H&E staining of the hippocampus of female 5xFAD Ctrl, 5xFAD *Fip200* cKO, and 5xFAD *Atg14* cKO mice. Enlarged images were shown in the middle for DG and on the right for CA1 region. **G** Mean ± SE of the DG area of female 5xFAD Ctrl, 5xFAD *Fip200* cKO, and 5xFAD *Atg14* cKO mice. CA1 Cornu Ammonis 1, GZ granular zone, SGZ subgranular zone. 5xFAD Ctrl = 5 mice, 5xFAD *Fip200* cKO = 5 mice, and 5xFAD *Atg14* cKO = 5 mice. One-way ANOVA with a Bonferroni correction was used for statistical analysis. ns no significance. Bar = 100 μm.

Our previous studies indicate that cKO of *Fip200* (essential for autophagy induction) or *Atg14* (essential for autophagy initiation) in microglia has no effect on the postnatal NSCs and neurogenesis in hippocampus of female 5xFAD mice at 4-month-old^63^. Nevertheless, it is not known whether loss of these genes in microglia will affect NSCs in the hippocampus and SVZ at a later stage of AD. We collected brain samples from female 5xFAD;*Fip200*^*CX3CR1*^ cKO (designated as 5xFAD *Fip200* cKO) mice and 5xFAD;*Atg14*^*CX3CR1*^ cKO (designated as 5xFAD *Atg14* cKO) mice along with their respective 5xFAD controls (5xFAD;*Fip200* flox/flox mice and 5xFAD;*Atg14* flox/flox mice) at 8-month-old (Fig. 7A). We first examined the degree of p62 aggregation in P2RY12^+^ microglia. We found that the deletion of *Atg5*, *Atg14*, and *Fip200* in hippocampal microglia caused more p62^+^ microglia and more p62^+^ puncta than in microglia from 5xFAD Ctrl mice (Fig. S3), suggesting a similar autophagy inhibition in these autophagy genes cKO microglia. Histological analyses indicated that the levels of Aβ plaques coverage in DG were comparable between 5xFAD Ctrl mice and 5xFAD *Atg14* cKO mice at 8-month-old (Fig. 7B, C). In comparison to these groups, 5xFAD *Fip200* cKO mice exhibited significantly fewer Aβ plaques (Fig. 7B, C). Moreover, X-34 staining confirmed more fibrillary Aβ plaques in the hippocampus of 5xFAD Ctrl mice and 5xFAD *Atg14* cKO than in 5xFAD *Fip200* cKO mice at 8-month-old (Fig. 7D, E). These results indicated that Fip200 deficiency in female microglia from older mice might accelerate the removal of Aβ plaques or reduce the generation of Aβ in the hippocampus. Despite the significant difference in Aβ plaque deposition, we detected more microglia in the DG of both 5xFAD *Fip200* cKO mice and 5xFAD *Atg14* cKO mice as compared to 5xFAD Ctrl mice (Fig. 7F, G). These results, along with the findings of *Atg5* deficiency increasing the number of microglia in 5xFAD mice from both sexes at 8-month-old (Fig. 3F, G), suggested that autophagy’s functions in inhibiting survival and/or promoting death of microglia were independent of Aβ accumulation and sex. Next, we used GFAP and SOX2 to label postnatal NSCs in the SGZ. We found a comparable number of NSCs in 5xFAD Ctrl mice, 5xFAD *Fip200* cKO mice, and 5xFAD *Atg14* cKO mice at 8-month-old (Fig. 8A, B). Further analyses indicate no significant differences in the number of DCX^+^ cells in female 5xFAD *Fip200* cKO and 5xFAD *Atg14* cKO mice as compared to their respective 5xFAD Ctrl mice (Fig. 8C, D). Moreover, we observed several Ki67^+^ proliferative cells in the SGZ of 5xFAD Ctrl mice, 5xFAD *Fip200* cKO mice and 5xFAD *Atg14* cKO mice (Fig. 8C, E) and we did not observe any Ki67^+^ DCX^+^ cells in the SGZ regions (Fig. 8C and data not shown). We examined the area of the CA1 and DG regions using H&E staining, with no differences observed across groups (Fig. 8F, G). These results suggested that Fip200-deficient and Atg14-deficient female microglia had no impact on the NSC’s maintenance and functions in AD hippocampus at 8-month-old.

### Deficiency of microglial *Fip200* and *Atg14* increased neuroblast formation without affecting NSC maintenance in the SVZ of 8-month-old female 5xFAD mice

**Fig. 9:**
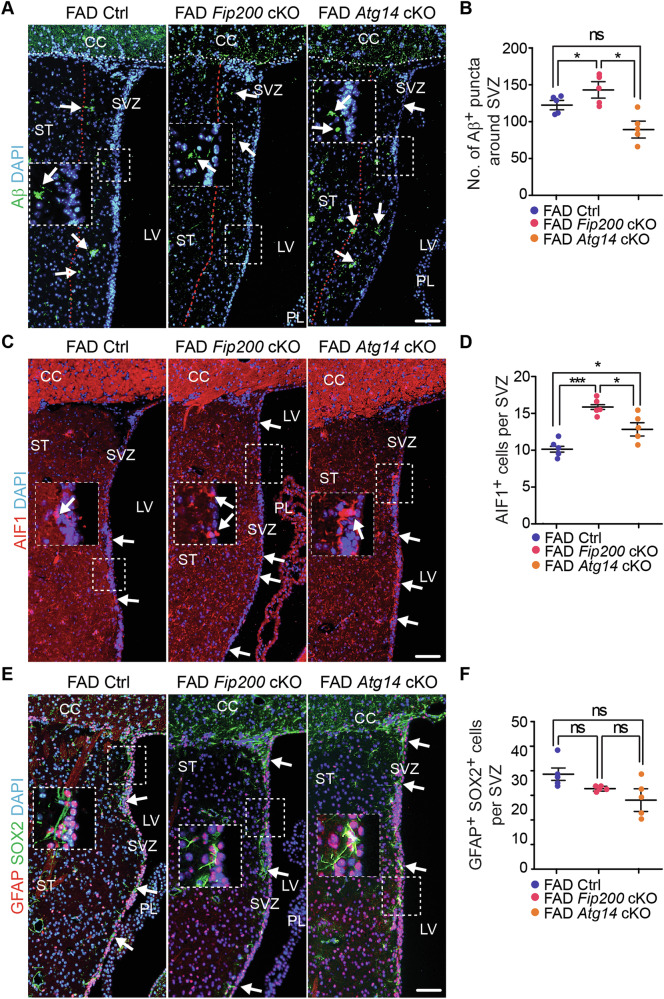
Deletion of Fip200 and Atg14 increased microglia in SVZ but had no impact on NSC maintenance of 8-month-old female AD mice. **A** IF of Aβ and DAPI in the striatum and SVZ of female 5xFAD Ctrl, 5xFAD *Fip200* cKO, and 5xFAD *Atg14* cKO mice at 8-month-old. Arrows indicated Aβ plaques. Boxed areas were shown in detail as insets. Red dotted lines indicated the regions 200 μm from the wall of lateral ventricle. **B** Mean ± SE of the number of Aβ puncta within 200 µm distance of SVZ in 5xFAD Ctrl, 5xFAD *Fip200* cKO, and 5xFAD *Atg14* cKO mice. **C** IF of AIF1 and DAPI in the SVZ of 5xFAD Ctrl, 5xFAD *Fip200* cKO, and 5xFAD *Atg14* cKO mice. Arrows indicated microglia. Boxed areas were shown in detail as insets. **D** Mean ± SE of the number of AIF1^+^ cells in the SVZ of 5xFAD Ctrl, 5xFAD *Fip200* cKO, and 5xFAD *Atg14* cKO mice. **E** IF of SOX2, GFAP, and DAPI in the SVZ of female 5xFAD Ctrl, 5xFAD *Fip200* cKO, and 5xFAD *Atg14* cKO mice. Arrows indicated NSCs. Boxed areas were shown in detail as insets. **F** Mean ± SE of the number of NSCs in the SVZ of female 5xFAD Ctrl, 5xFAD *Fip200* cKO, and 5xFAD *Atg14* cKO mice. CC corpus callosum, LV lateral ventricle, PL plexus, ST striatum, SVZ subventricular zone. 5xFAD Ctrl = 5 mice, 5xFAD *Fip200* cKO = 5 mice, and 5xFAD *Atg14* cKO = 5 mice. One-way ANOVA with a Bonferroni correction was used for statistical analysis. ns no significance, *: p < 0.05, ***: p < 0.001. Bar = 100 μm.

**Fig. 10:**
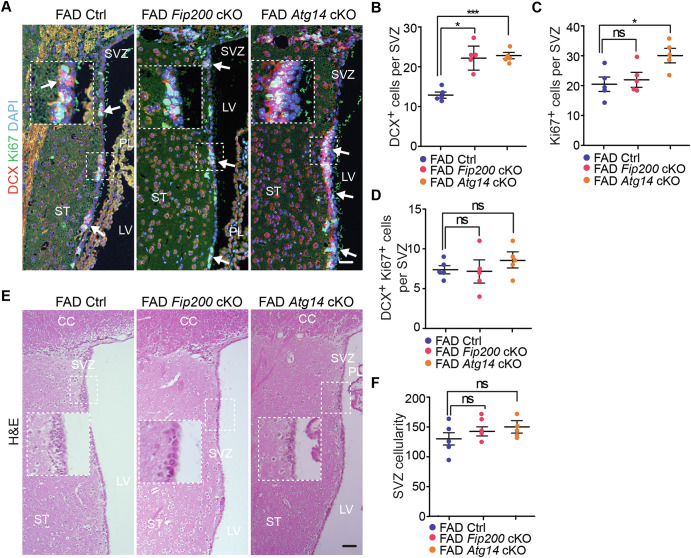
Deletion of Fip200 and Atg14 increased neuroblast generation in SVZ of female AD mice at 8-month-old. **A** IF of DCX, Ki67, and DAPI in SVZ of female 5xFAD Ctrl, 5xFAD *Fip200* cKO, and 5xFAD *Atg14* cKO mice at 8-month-old. Arrows indicated DCX^+^ Ki67^+^ neuroblasts. Boxed areas were shown in detail as insets. Mean ± SE of the number of DCX^+^ cells (**B**), Ki67^+^ cells (**C**), and DCX^+^ Ki67^+^ cells (**D**) in SVZ of 5xFAD Ctrl, 5xFAD *Fip200* cKO, and 5xFAD *Atg14* cKO mice at 8-month-old. **E** H&E staining of SVZ in 5xFAD Ctrl, 5xFAD *Fip200* cKO, and 5xFAD *Atg14* cKO mice. Boxed areas were shown in detail as insets. **F** Mean ± SE of the number of SVZ cells in 5xFAD Ctrl, 5xFAD *Fip200* cKO, and 5xFAD *Atg14* cKO mice. CC corpus callosum, LV lateral ventricle, PL plexus; ST: striatum, SVZ subventricular zone. 5xFAD Ctrl = 5 mice, 5xFAD *Fip200* cKO = 5 mice, and 5xFAD *Atg14* cKO = 5 mice. One-way ANOVA with a Bonferroni correction was used for statistical analysis. ns no significance, *: p < 0.05, ***: p < 0.001. Bar = 100 μm.

Next, we examined the SVZ region in female 5xFAD Ctrl, 5xFAD *Fip200* cKO, and 5xFAD *Atg14* cKO mice at 8-month-old. We quantified the Aβ^+^ dots within an area of 200 μm from the wall of LV to the striatum. We detected more Aβ^+^ dots in the regions of the SVZ in 5xFAD *Fip200* cKO mice compared to 5xFAD Ctrl mice (Fig. 9A, B). However, loss of microglial *Atg14* had no effect on the amount of Aβ^+^ material in the SVZ in 5xFAD females (Fig. 9A, B). Similar to the hippocampus, loss of either microglial *Fip200* or *Atg14* increased the number of microglia in the SVZ of female 5xFAD mice (Fig. 9C, D). We next performed double staining of GFAP and SOX2 to label NSCs in the SVZ. We found a trend for modest decrease in the number of NSCs in SVZ of female 5xFAD *Fip200* cKO mice and 5xFAD *Atg14* cKO mice compared to 5xFAD Ctrl mice at 8-month-old (Fig. 9E, F). To examine the newly formed neurons, we performed double staining of DCX with Ki67. Interestingly, we found more DCX^+^ cells in SVZ of 5xFAD *Fip200* cKO mice and 5xFAD *Atg14* cKO mice than in 5xFAD Ctrl mice (Fig. 10A, B). The number of Ki67^+^ progenitors was significantly higher in 5xFAD *Atg14* cKO SVZ than in 5xFAD Ctrl SVZ, which showed a comparable number of proliferative cells as in 5xFAD *Fip200* cKO mice (Fig. 10A, C). Nevertheless, we detected no difference in DCX^+^ Ki67^+^ immature neuron number between groups (Fig. 10A, D). H&E staining revealed only a modest trend toward reduced SVZ tissue thickness in 5xFAD female mice lacking microglial *Fip200* or *Atg14* (Fig. 10E, F). Taken together, this data suggested that even though loss of *Fip200* and *Atg14* in microglia promoted the expansion of neuroblasts in the SVZ of AD mice, these two autophagy proteins in microglia had no impact on the maintenance of postnatal NSCs in female AD mice at 8-month-old.

## Discussion

In this study, we compared the functions of *Atg5* in male and female microglia for their gaining of DAM gene signatures in the AD hippocampus at an early disease stage. Our results indicated that female microglia, but not male microglia relied on Atg5 to obtain DAM gene signatures to protect postnatal neurogenesis in mice in AD progression. The DAM promoting features and protective functions of Atg5 in female microglia seemed to be independent of its autophagy functions, as other canonical autophagy genes of *Fip200* and *Atg14* in female microglia were not involved in the maintenance and neurogenesis of NSCs in AD mice, even at advanced disease stages.

DAM has been proposed to limit AD progression by clearing plaques, cellular debris, and other threats^51,53,68,69^. DAM depends on the well characterized Trem2-ApoE pathway for full maturation^53,70,71^. Nevertheless, the molecular mechanisms underlying the sex differences in regulating DAM are not well understood. DAM markers are more enriched in aged female microglia than in aged male microglia, possibly because of female-biased activation of Trem2 in old microglia^72^. The more significantly decreased mRNA levels of *Trem2* and *Apoe* as well as protein level of Trem2 in female than in male *Atg5* cKO microglia suggested critical functions of Atg5 for DAM activation in female AD hippocampus. It is possible that the difference of metabolism in male and female microglia could be a mechanism for the regulation of DAM by Atg5. Old female microglia exhibit increased glycolysis compared to old male microglia^73^ and microglia from female APP/PS1 mice also shift their metabolism towards glycolysis^74^. Atg5 deficiency impairs glucose metabolism by decreasing surface expression of Glut1 through its noncanonical roles in membrane atg8ylation in retromer assembly and function^75^. We speculate that female *Atg5* cKO microglia fail to sustain higher glycolytic activity because of increased demand in DAM activation. It has also been noticed that aged female microglia display robust senescent phenotypes which is less evident in aged male microglia^72^. Autophagy in general prevents cellular senescence. Choi et al. reveal a role of Atg7 in microglia to prevent senescence^62^ which is detrimental in the AD brain^76^. Thus, our data suggested another possibility that Atg5 deficiency specifically accelerated senescence in female microglia to inhibit neurogenesis at the early stage of AD.

Our results showed that at 8-month-old, the generation of new neurons in the hippocampus was scant and was comparable between 5xFAD Ctrl mice and 5xFAD *Atg5* cKO mice from both sexes (Fig. 4). Similar findings were observed in the hippocampus of female 5xFAD *Atg14* cKO and 5xFAD *Fip200* cKO mice at the same age (Fig. 8). These findings were consistent with the dramatic decrease in the rate of hippocampal neurogenesis, which drops by around 80% from 2 to 8 month of age in the rodent brain^77^. To our surprise, the number of DCX^+^ cells in the SVZ significantly increased in 5xFAD *Atg5* cKO mice as well as in female 5xFAD *Fip200* cKO and 5xFAD *Atg14* cKO mice when compared with their respective controls at 8-month-old (Figs. 6 and 10). The distinct functions of autophagy genes in microglia for neurogenesis in the hippocampus and SVZ might reflect microglial regional heterogeneity^78^. SVZ microglia are more of an ameboid shape, and hippocampal microglia are usually ramified. The higher phagocytotic activity for dead neural progenitors and neuroblasts in SVZ microglia^79^ is critical for the neurogenesis function of the adult SVZ. Trem2 expression level is high in hippocampal microglia but low in microglia from the SVZ^80^ which suggests that hippocampal microglia depend more on Atg5 to maintain Trem2 expression for DAM in AD neurogenesis. Future studies will reveal the mechanisms of Atg5/autophagy in regional microglial heterogeneity for adult neurogenesis in AD mice.

Microglia are known to be relatively long-living cells that show gradual turnover with limited self-renewal capacity in the brain^81,82^. *Atg5* cKO in microglia has no impact on the cell number and the development of experimental autoimmune encephalomyelitis^83^. In this study, we found that the deletion of autophagy genes *Atg5*, *Atg14*, and *Fip200* in microglia significantly increased their number in AD hippocampus at 8-month-old, and the fold of increase was more prominent in male 5xFAD *Atg5* cKO microglia (Figs. 3 and 7). The increase of microglia number was irrelevant to sex as it happened in both male and female autophagy defective mice. Atg5 is a key factor for maintaining the balance between autophagy and apoptosis^84^. In other cell types, a previous study has shown that Atg5 promotes the apoptosis of cancer cells independent of autophagy^85^. Atg5 promotes the apoptosis of macrophages by enhancing the Fas-FasL signaling pathway after malaria infection, which is mechanically distinct from Atg5-mediated apoptosis of cancer cells^86^. Autophagic death of NSCs mediates chronic stress-induced decline of adult hippocampal neurogenesis and cognitive deficits^87^. Even though autophagy deficiency in microglia has no impact on their survival or apoptosis in normal development or neuroinflammatory disease, the consistent role of *Atg5*, *Atg14*, and *Fip200* in AD microglial survival suggest autophagy as a decisive process for cell death^88^ in these innate immune cells in AD progression. Another possibility is that autophagy inhibition increases the proliferation of microglia in the AD hippocampus. However, we did not find significant difference in the proliferation of DG cells (Ki67^+^) from 5xFAD Ctrl mice and 5xFAD autophagy cKO mice (Figs. 4 and 8), indicating autophagy’s functions in restricting microglia number in AD hippocampus are not likely through increased cell proliferation. Since we used AIF1 to stain cells from myeloid lineages, however, it was uncertain whether these AIF1^+^ cells were microglia or if some of them were from infiltrating myeloid cells, such as macrophages. In our future studies, detailed lineage tracing experiments will help to determine the origins of the increased AIF1^+^ myeloid cells in the 5xFAD hippocampus without an autophagy gene.

Even though the functions and mechanisms of Atg5 in autophagy have been well studied, we admit the limit of our current study for not revealing additional molecular mechanisms of Atg5 in regulating DAM gene signatures in a sex-specific manner. Previous studies indicate that *Toxoplasma gondii* infection in human cells increases the interaction of Atg5 with glycolytic enzymes (i.e., ALODOA, LDHA, and PGK1)^89^. These glycolytic proteins are not reported to interact with Fip200 or Atg14. It is possible that the regulation of these glycolytic proteins might contribute to the function of Atg5 in regulating DAM gene signatures in AD hippocampus. We will warrant future research in elucidating the canonical and non-canonical functions of Atg5 in microglia to clarify the molecular mechanisms for the sex differences in AD neurogenesis.

In summary, our study using microglia specific mouse models deleting different autophagy genes revealed an essential role of Atg5 in female microglia to maintain DAM gene signatures for postnatal neurogenesis in hippocampus of AD brain. This study also implies that targeting Atg5 has the potential to treat and/or prevent neurogenesis loss in AD progression in female patients.

## Materials and methods

### Animals

WT, 5xFAD, *Atg5* flox/flox, *Atg14* flox/flox, *Fip200* flox/flox, CX3CR1^CreERT2^;*Atg5* flox/flox;5xFAD, CX3CR1^CreERT2^;*Atg14* flox/flox;5xFAD, CX3CR1^CreERT2^;*Fip200* flox/flox;5xFAD mice with B6 background were described as before^63^. *Atg5* flox/flox, and *Atg14* flox/flox mice were gifted from Dr. Herbert Virgin’s lab at the Washington University, *Fip200* flox/flox mice were generated in Dr. Jun-Lin Guan’s lab at the University of Cincinnati, CX3CR1^CreERT2^ mouse was purchased from Jackson lab (JAX, 021160). 5xFAD Ctrl, CX3CR1^CreERT2^;*Atg5* flox/flox;5xFAD, CX3CR1^CreERT2^;*Atg14* flox/flox;5xFAD, and CX3CR1^CreERT2^;*Fip200* flox/flox;5xFAD mice and their respective 5xFAD Ctrl mice were intraperitoneally injected with 1 mg tamoxifen (TAM) at 1 month old for 4 times. Mice were housed and handled according to local, state, and federal regulations. All experimental procedures were conducted according to the guidelines of Institutional Animal Care and Use Committee (IACUC) at University of Cincinnati (21-06-21-02).

### Antibodies and reagents

Primary antibodies used were mouse anti-Aβ (Biosensis, M-1742-50-B), anti-GFAP (Cell Signaling Technology, 3670), anti-P2RY12 (BioLegend, 848001 and 848004), anti-ATG5 (BioLegend, 847401); rabbit anti-AIF1 (WAKO, 019-19741), anti-MKI67/Ki67 (Spring Bioscience, 151213), anti-GFAP (DAKO, M0761), anti-p62 (Enzo, BML-PW9860), anti-SOX2 (Millipore, AB5603); rat anti-ITGAM/CD11b (BD Pharmingen, 557296), anti-MKI67 (BioLegend, 151213), anti-TREM2 (R&D Systems, MAB17291); and guinea pig anti-DCX (Millipore, AB2253). Secondary antibodies were goat anti-rabbit IgG-FITC (Jackson Immunology, 111-095-003), goat anti-rabbit IgG-Alexa Fluor (Jackson Immunology, 111-586-003), goat anti-mouse IgG-FITC (Jackson Immunology, 115-095-003), goat anti-mouse IgG-Texas Red (Jackson Immunology, 115-295-003), goat anti-mouse IgM-Rhodamine (Jackson Immunology, 115-025-020), goat anti-guinea pig IgG-Texas Red (Jackson Immunology, 106-585-003), goat anti-mouse IgG-HRP (Jackson Immunology,115-035-00.), and goat anti-rabbit IgG-HRP (Jackson Immunology, 111–035-144). Tamoxifen (T5648) and X-34 (SML1954) were purchased from Sigma (St Louis, MO).

### Histology and immunofluorescence (IF)

Mice were euthanized using CO_2_, and the brain was harvested during necropsy. Fixation was conducted for 16 h at 4 °C using 4% (w:v) freshly made, pre-chilled PBS (Invitrogen, 10010023) buffered paraformaldehyde/PFA. The brain tissues were all sagittally separated into two hemispheres through the midline and one hemisphere was embedded in paraffin, sectioned at 5 μm. Slides from histologically comparable positions (triangular lateral ventricle with intact rostral migratory stream) were stained with hematoxylin and eosin (H&E) for routine histological examination or left unstained for immunofluorescence (IF). H&E-stained sections were examined under a BX41 light microscope (Olympus America, Inc., Center Valley, PA), and images were captured with an Olympus digital camera (model DP75) using DP Controller software (Version 1.2.1.10 8). For IF, unstained tissues were first deparaffinized in 3 washes of xylene (3 min each) and then were rehydrated in graded ethanol (EtOH) solutions (100 × 3 times, 95, 70, 50, and 30%, 1 min each). After heat-activated antigen retrieval (Retriever 2000, PickCell Laboratories B.V., Amsterdam, Holland) according to the manufacturer’s specifications, sections were treated with Protein Block Serum Free (Agilent, X090930-2) at room temperature for 10 min. Slices were then incubated with the primary antibodies at 4 °C for 16 h in a humidified chamber, washed in PBS for 3 times (5 min each) and incubated with the 1:200 secondary antibodies for 1 h at room temperature. After incubation with secondary antibodies and a wash in PBS 3 times (5 min each), nuclei were stained with DAPI and mounted with Vectashield mounting medium (Vector Laboratories, H-1200-10). Digital photography was carried out as described previously^90^. For staining of Aβ plaques, X-34 diluent (40% EtOH, pH 10) was prepared by adding 0.5 ml stock solution of X-34 (5 mM) with 20 mL of EtOH and 29.5 mL of dH_2_O. The we added 1 µL of 5 M NaOH dropwise until the solution reached pH 10. The slides were stained in 25 µM X-34 staining solution for 30 min at room temperature in the dark. Then, the slides were washed three times in dH_2_O and further incubated for 2 min in the differentiation buffer (50 mM NaOH, 80% EtOH). The slides were rinsed three times in dH_2_O before mounted for IF imaging.

### Percoll gradient isolation and FACS for enriched microglia

The method for isolation of microglia was as what we described before^66^. In brief, dissected cortex and hippocampus were passed through a 70-μm cell strainer. Homogenates were centrifuged at 600 × *g* for 6 min. Supernatants were removed, and cell pellets were resuspended in 70% isotonic Percoll (GE Healthcare, 17-5445-01). A discontinuous Percoll density gradient was layered as follows: 70%, 30%, and 0% isotonic Percoll. The gradient was centrifuged for 20 min at 2000 × *g*, and enriched microglia were collected from the interphase between the 70% and 30% Percoll layers. Enriched microglia were labeled with antibodies for flow cytometry and sorted based on the detection of CD11b and P2RY12 using a Bio-Rad S3e cytometer/cell sorter (Bio-Rad, 12007058). All data were analyzed using FlowJo v10 software (Tree Star Inc.).

### Real time PCR

Total RNA was isolated from sorted microglia using a Single Cell RNA Purification Kit (Norgen Biotek Corp., 51800) according to the user manual. Reverse transcription complementary DNA (cDNA) was synthesized with iScript cDNA Synthesis Kit (Bio-Rad, 1708891). Real-time PCR was performed with iQ SYBR Green Supermix Kit (Bio-Rad, 170-8880). Expression values were normalized to *Actb/β-actin*. The primers were obtained from PrimerBank (https://pga.mgh.harvard.edu/primerbank/) unless specific references were cited. The specificity of primers was validated via dissociation curves.

### Statistical analysis

Lengths, areas, and the number of cells from comparable sections were quantified using the ImageJ software package. Statistical significance was evaluated by one-way ANOVA with Bonferroni correction and Student’s t-test with *p* < 0.05 using Graph Pad Prism (Version 7.0). The number of animals used for quantification is indicated in the figure legends.

## Supplementary information

Supplementary Figures

## Data Availability

No datasets were generated or analyzed during the current study.
