# Supplementary material for: Atg5 in microglia regulates sex-specific effects on postnatal neurogenesis in Alzheimer’s disease
Source: NPJ Aging. 2025 Mar 16;11(1):18. doi: 10.1038/s41514-025-00209-0 (PMC11911432; doi:10.1038/s41514-025-00209-0)
Supplement: Supplementary file 1 — Supplementary Figures [file 41514_2025_209_MOESM1_ESM.pdf]

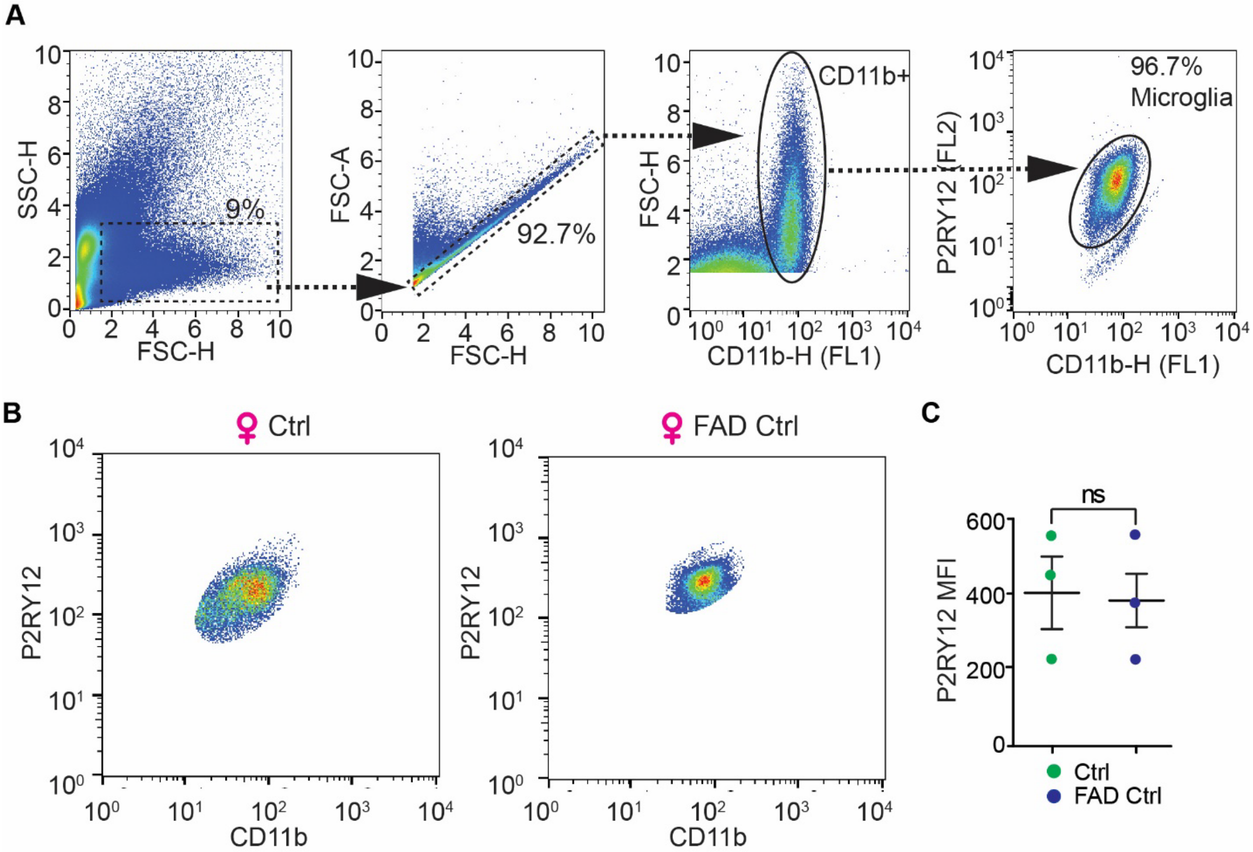
A
SSC-H
FSC-H
9%
FSC-A
FSC-H
92.7%
FSC-H
CD11b-H (FL1)
CD11b+
P2RY12 (FL2)
CD11b-H (FL1)
96.7%
Microglia
B
♀ Ctrl
P2RY12
CD11b
♀ FAD Ctrl
P2RY12
CD11b
C
ns
P2RY12 MFI
Ctrl
FAD Ctrl

Figure S1

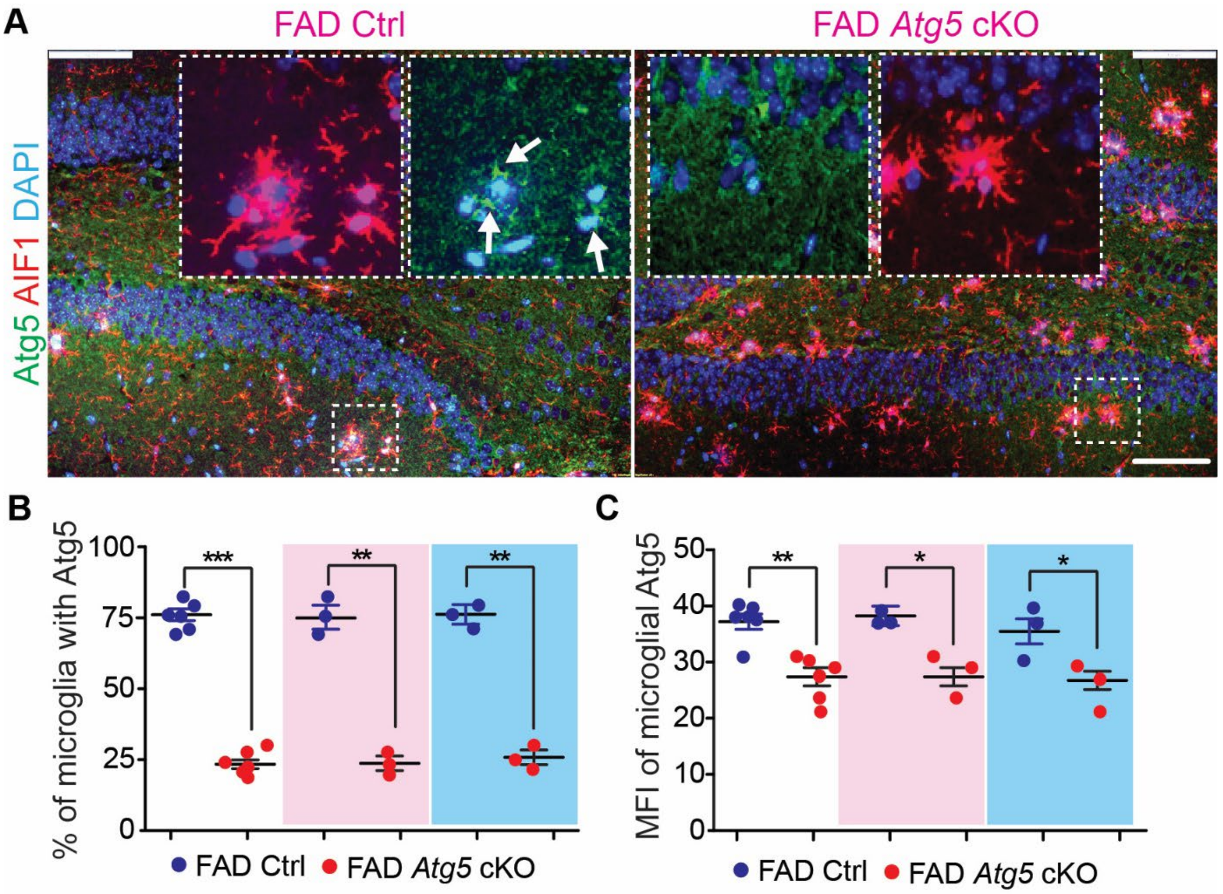
A
FAD Ctrl
FAD Atg5 cKO
Atg5 AIF1 DAPI
B
% of microglia with Atg5
100
75
50
25
0
***
**
**
FAD Ctrl
FAD Atg5 cKO
C
MFI of microglial Atg5
50
40
30
20
10
0
**
*
*
FAD Ctrl
FAD Atg5 cKO

Figure S2

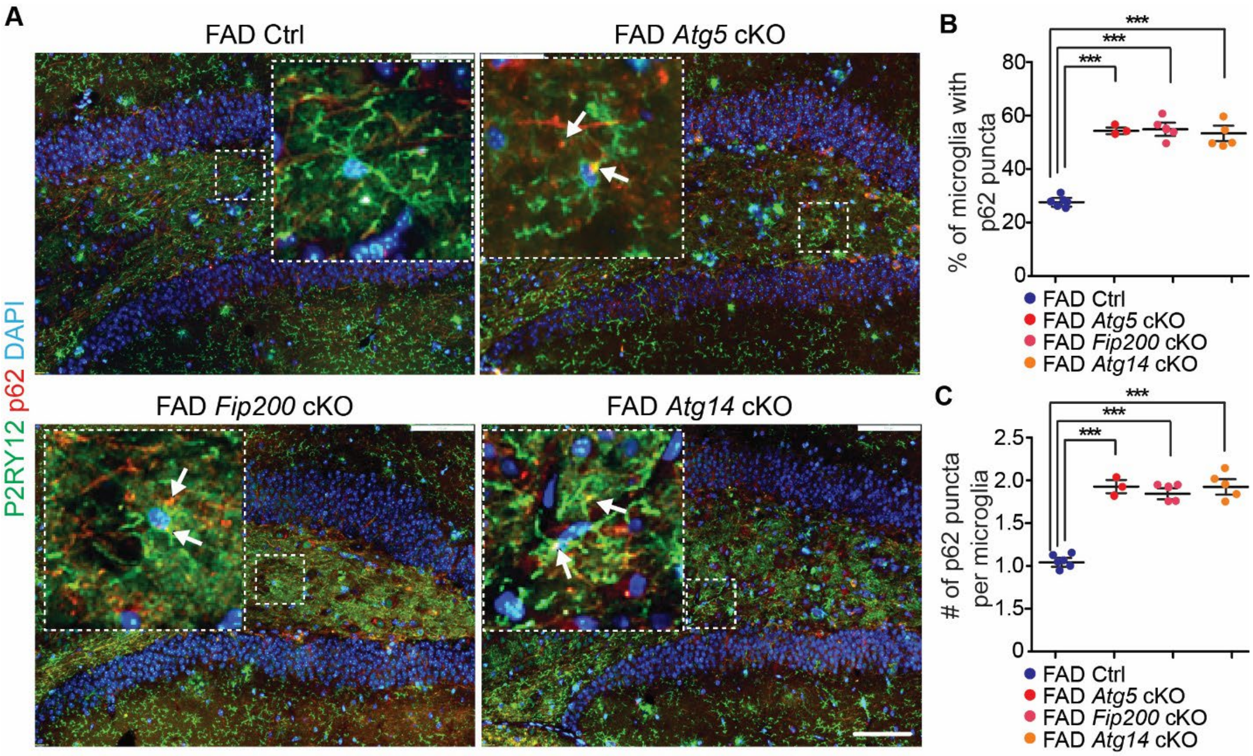

A
FAD Ctrl
FAD *Atg5* cKO
FAD *Fip200* cKO
FAD *Atg14* cKO
P2RY12 p62 DAPI
B
% of microglia with p62 puncta
80
60
40
20
0
***
***
***
FAD Ctrl
FAD *Atg5* cKO
FAD *Fip200* cKO
FAD *Atg14* cKO
C
# of p62 puncta per microglia
2.5
2.0
1.5
1.0
1.5
0
***
***
***
FAD Ctrl
FAD *Atg5* cKO
FAD *Fip200* cKO
FAD *Atg14* cKO

Figure S3

Figure S1. Gating microglia and comparison of P2RY12 in control and 5xFAD Ctrl hippocampus. (A) Gating strategy for microglia isolation. (B) Flow cytometry analyses of the surface expression of CD11b and P2RY12 on isolated hippocampal microglia from female control and 5xFAD Ctrl mice at 4-month-old. (C) Mean $\pm$ SE of the MFI of P2RY12 on microglia from female control and 5xFAD Ctrl mice at 4-month-old. Control mice = 3 and 5xFAD Ctrl mice = 3. The Student's t-test was used for statistical analysis. ns: no significance.

Figure S2. Deletion of Atg5 in 5xFAD *Atg5* cKO microglia. (A) IF of AIF1, Atg5, and DAPI of hippocampus in 5xFAD Ctrl and 5xFAD *Atg5* cKO mice at 8-month-old. Arrows indicated $Atg5^+$ $AIF1^+$ cells. Boxed areas were shown in detail as insets. (B and C) Mean $\pm$ SE of the percentage of $Atg5^+$ $AIF1^+$ cells of all $AIF1^+$ cells (B) and MFI of Atg5 in microglia (C) from hippocampus of 5xFAD Ctrl and 5xFAD *Atg5* cKO mice. 5xFAD Ctrl = 6 mice (3 male and 3 female) and 5xFAD *Atg5* cKO = 6 mice (3 male and 3 female) from both sexes. The Student's t-test was used for statistical analysis. *: $p < 0.05$; **: $p < 0.01$; ***: $p < 0.001$. Bar = 100 μm.

Figure S3. Autophagy deficiency in microglia of 5xFAD *Atg5* cKO, 5xFAD *Fip200* cKO, and 5xFAD *Atg14* cKO mice. (A) IF of P2RY12, p62, and DAPI of hippocampus in female 5xFAD Ctrl, 5xFAD *Atg5* cKO, 5xFAD *Fip200* cKO, and 5xFAD *Atg14* cKO mice at 8-month-old. Arrows indicated $p62^+$ puncta in $P2RY12^+$ cells. Boxed areas were shown in detail as insets. (B) Mean $\pm$ SE of the percentage of $P2RY12^+$ cells with $\geq$ 2 $p62^+$ puncta of all $P2RY12^+$ cells in hippocampus of 5xFAD Ctrl, 5xFAD *Atg5* cKO, 5xFAD *Fip200* cKO, and 5xFAD *Atg14* cKO mice. (C) Mean $\pm$ SE of the number of $p62^+$ puncta per $P2RY12^+$ cell in the hippocampus of 5xFAD Ctrl, 5xFAD *Atg5* cKO, 5xFAD *Fip200* cKO, and 5xFAD *Atg14* cKO mice. 5xFAD Ctrl = 6 mice, 5xFAD *Atg5* cKO = 3 mice, 5xFAD *Fip200* cKO = 5 mice, and 5xFAD *Atg14* cKO = 5 mice. One-way ANOVA with a Bonferroni correction was used for statistical analysis. ***: $p < 0.001$. Bar = 100 μm.
